# Co-Designing a Collaborative Chronic Care Network (C3N) for Inflammatory Bowel Disease: Development of Methods

**DOI:** 10.2196/humanfactors.8083

**Published:** 2018-02-22

**Authors:** Michael Seid, George Dellal, Laura E Peterson, Lloyd Provost, Peter A Gloor, David Livingstone Fore, Peter A Margolis

**Affiliations:** ^1^ Pulmonary Medicine Department of Pediatrics Cincinnati Children's Hospital Medical Center Cincinnati, OH United States; ^2^ James M Anderson Center for Health Systems Excellence Department of Pediatrics Cincinnati Children's Hospital Medical Center Cincinnati, OH United States; ^3^ Associates in Process Improvement Austin, TX United States; ^4^ Center for Collective Intelligence Massachusetts Institute of Technology Cambridge, MA United States; ^5^ Catabolic Design Oakland, CA United States

**Keywords:** chronic disease, pediatrics, health care delivery, quality improvement

## Abstract

**Background:**

Our health care system fails to deliver necessary results, and incremental system improvements will not deliver needed change. Learning health systems (LHSs) are seen as a means to accelerate outcomes, improve care delivery, and further clinical research; yet, few such systems exist. We describe the process of codesigning, with all relevant stakeholders, an approach for creating a collaborative chronic care network (C3N), a peer-produced networked LHS.

**Objective:**

The objective of this study was to report the methods used, with a diverse group of stakeholders, to translate the idea of a C3N to a set of actionable next steps.

**Methods:**

The setting was ImproveCareNow, an improvement network for pediatric inflammatory bowel disease. In collaboration with patients and families, clinicians, researchers, social scientists, technologists, and designers, C3N leaders used a modified idealized design process to develop a design for a C3N.

**Results:**

Over 100 people participated in the design process that resulted in (1) an overall concept design for the ImproveCareNow C3N, (2) a logic model for bringing about this system, and (3) 13 potential innovations likely to increase awareness and agency, make it easier to collect and share information, and to enhance collaboration that could be tested collectively to bring about the C3N.

**Conclusions:**

We demonstrate methods that resulted in a design that has the potential to transform the chronic care system into an LHS.

## Introduction

### Background and Rationale

It has long been known that within the health care system, patients across care settings are prescribed half of indicated care [1-3] and follow through on half of what is prescribed [4]; translating interventions into practice takes too long [5], and research is too expensive, too slow, and does not reflect the needs of patients seen in real-world settings. This is not because of a lack of will or ideas but rather, to the absence of a system in which the efforts and ideas of all stakeholders are translated to improvement.

What if we could create a vastly better chronic illness care system by harnessing the inherent motivations and collective intelligence of patients and families, clinicians, and researchers, so that all could collaborate, at scale, to improve health?

This provocation was the seed for the collaborative chronic care network (C3N) project [6]. The C3N model reflects the Institute of Medicine’s learning health system (LHS) [7] in which health care, improvement, and research are purposefully integrated, but extends that model, via network-based or peer production [8], to all stakeholders. The C3N model reflects scientific advances over the last 20 years in cooperative behavior [9], collective intelligence [10], and organizational architecture for innovation [11], which point to a fundamental principle: people are, by and large, cooperative and generous. The design of systems, including chronic care systems, can hinder or facilitate expression of these impulses.

Design processes are widely used to create and modify products, services, and systems [12]. The purpose of design is to imagine new and better ways to match products, services, or systems with user contexts and goals [13]. Cooperative design, often shortened to codesign [14], refers to actively involving all stakeholders in every stage of the design process, which ensures that the end product meets the needs of all stakeholders. In this way, the design process and the design results become a reinforcing loop, aligning stakeholders and facilitating collaborative action to achieve that design.

### Objective

The objective of this study was to report the methods used, with a diverse group of stakeholders, to translate the idea of a C3N to a set of actionable next steps. Although we have previously described elements of the C3N model [6,15], the use of goal-directed design [16], and the formation of a collaborative open-innovation network [17], the unique contribution of this report is describing the process of codesigning—with representatives from all relevant stakeholders and using the idealized design process—an approach that has the potential to transform the chronic care system. We use the case of ImproveCareNow, a learning network to improve health, care, and costs for pediatric patients with inflammatory bowel disease (IBD), as the model case for these methods.

## Methods

### Human Subjects Protection

This research was reviewed and approved by the institutional review board at the corresponding author’s home institution.

### Setting and Population

ImproveCareNow was launched in 2007 to improve health, care, and costs for children with Crohn’s disease and ulcerative colitis; together, IBD [6]. Known previously as PIBDNet, ImproveCareNow originally included nine care centers that used a modified Breakthrough Series model [18] to create a quality improvement (QI) network focused on improving remission rates for their patients [19]. ImproveCareNow initially involved clinicians, without significant involvement of patients, families, or other stakeholders. At the time of the design process (January 2010-July 2011), the ImproveCareNow network had grown to 24 care centers, with data on 2500 patients from 7500+ visits.

The C3N project, funded by an National Institutes of Health (NIH) Transformative Research Awards, aimed to design, prototype, and pilot a C3N—a potentially transformative system for chronic care. The C3N project partnered with ImproveCareNow to help it transform itself from a QI network into a C3N

The C3N team that led this work was composed of a pediatrician and epidemiologist, a behavioral and social scientist, improvement experts, designers, an expert in collective intelligence, and project management staff. Subject matter experts were integrated into the leadership team: pediatric IBD patients, parents of patients with IBD, pediatric gastroenterologists, and other pediatric IBD clinicians. All members of the leadership team also participated in the design process.

### Design Process Participants

The initial design meeting included youth with IBD, parents of children and youth with IBD, pediatric gastroenterologists, nurses and other clinicians, and a variety of other experts including designers, technologists, artists, QI specialists, social scientists, intellectual property experts, and community organizers.

Participants in this initial meeting were recruited from ImproveCareNow by identification of individuals through literature and Internet search, as well as by snowball sampling, in which existing participants are asked to nominate other potential participants.

### Design Process

**Figure 1:**
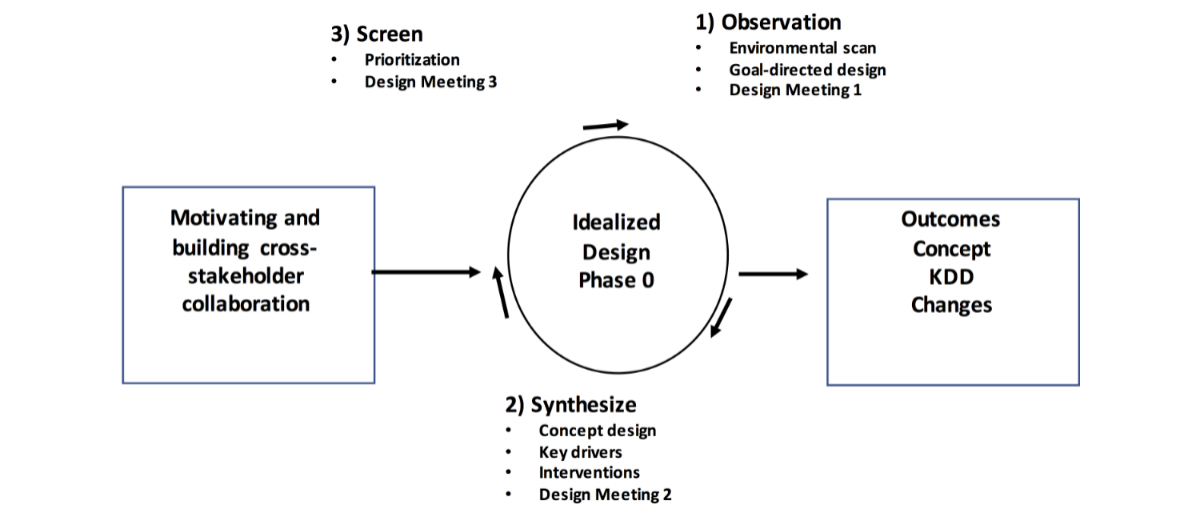
Design process used. KDD: key driver diagram.

**Figure 2:**
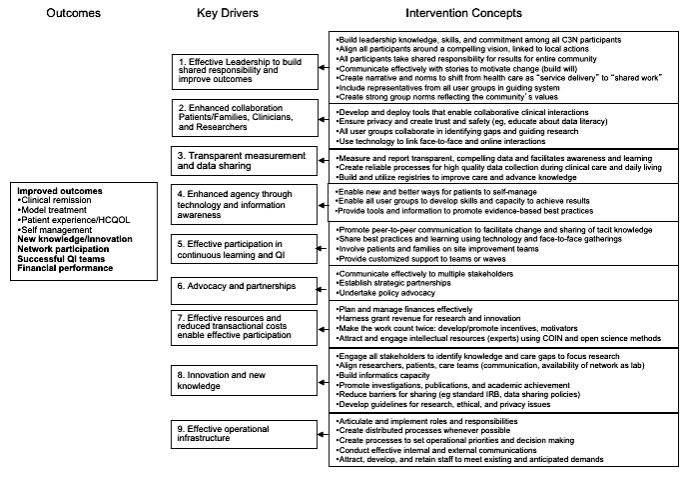
ImproveCareNow collaborative chronic care network (C3N) key driver diagram. QI: quality improvement.

We combined several approaches to design the C3N. These are illustrated in Figure 1. We used theories from the leadership of social movements and collective intelligence to motivate and build cross-stakeholder collaboration, and the idealized design process (specifically, phase 0), including observation, synthesis, and screening, to produce outcomes measures, the design concept, a key driver diagram (KDD; see Figure 2), and potential changes. These are further detailed below.

### Motivating and Building Cross-Stakeholder Collaboration

We used theories from leadership of social movements [20] to motivate and build cross-stakeholder collaboration. To build motivation, we created forums for patients, parents, and clinicians to share what Ganz calls a public narrative—stories that weave together values and emotion to cause action. We began each design meeting, for example, by having patients and parents share their public narrative. We also developed motivation for cross-stakeholder collaboration by creating a common vision of an idealized state (Idealized Design section, below).

We built cross stakeholder collaboration by emphasizing the enormity of the challenge and the need for everyone’s expertise and effort using specific messaging around urgency, hope, and self-efficacy to encourage participation (eg, *You can make a difference*) and solidarity (eg, *be part of the solution* [20]). We made an effort to promote diversity and inclusion by covering participant travel expenses, refraining from jargon and acronyms, and enabling remote participation.

We set out to create a small team of intrinsically motivated innovators working voluntarily together to realize innovative ways to tackle the thorny problem of systemic improvement of chronic illness care [10]. We developed a social media presence; contacted people directly via phone, in person, or email; and invited those interested to an online community of innovators using a private social networking platform. We looked for people who had implemented creative workarounds to the systems’ barriers (in other words, who had hacked the health care system) and who were eager to collaborate with likeminded others. We responded quickly and substantively to potential solutions and connected together people working on similar problems. These efforts were augmented by webinars where stakeholders shared their perspectives and relevant work and other webinars where ideas from different disciplines were integrated into the overall design [17].

There are inherent power gradients in health care, and we managed these by explicitly acknowledging this dynamic, by privileging patient and family voices (eg, framing the context for all design meetings by having a patient or parent share their public narrative), and by active facilitation to ensure that patient and family voices were included.

### Idealized Design

We used the idealized design process [21], a systematic process for creating and implementing new ideas through five steps (design, prototype, pilot, implementation, scale-up, and spread). Phase 0, the design phase, is an iterative process of observation, synthesis, and screening. It is focused on generating new ideas that could lead to a fundamental redesign to better meet the needs of users of the system. The 10-month iterative process consisted of interactions both synchronous (conference calls, webinars, and face-to-face meetings) and asynchronous (email and a private social media site). This process was punctuated by three design meetings, the objectives of which are provided in Textbox 1.

#### Observation—Environmental Scan, Goal-Directed Design, and Needs of the Users

Observation is the primary method for understanding patient needs and for generating ideas to meet those needs [12]. We used three techniques for observation—an environmental scan, goal-directed design, and understanding the needs of users. Innovations emerge from the inferences drawn from these observations.

We conducted an environmental scan to identify ideas and concepts that could fulfill user needs. We used a broad set of tools including key informant interviews, literature review, Internet searches, and group discussions.

We used goal-directed design—described in-depth elsewhere [16]—to understand human needs, as well as ideas that may satisfy those needs within a complex system. Goal-directed design begins with ethnographic and synthesis methods that generate *personas* —research-based composites of potential users of the new system—and *scenarios* that depict personas realizing their goals through interacting with the new system.

Understanding user needs and the current state informs the development of high level outcome measures. During design meeting 1, participants predicted the needs and goals of patients or families, clinicians, and researchers. These were synthesized to create measurement concepts and then outcome measures to assess the ability of a C3N to achieve its aims, centered on human needs, during the testing phase.

#### Synthesizing—Concept Design, Key and Secondary Drivers, and Innovations

Design synthesis is the abductive process of organizing and manipulating observations, data, and ideas into a coherent whole, both synthesizing observations into interventions and synthesizing interventions into a concept design. Observations were synthesized into a conceptual framework (a high-level description of what a C3N is and ought to do), a set of *key drivers* that must be in place to change the outcomes, and a set of intervention concepts, called *secondary drivers*, that might bring about these key drivers [22].

During design meeting 2, participants used the secondary drivers and personas to generate scenarios and a set of innovations (prototypes) that could be tested for their ability to change outcomes, whether individually or in combination. Participants also screened and elaborated on the KDD, metrics and targets, and possible innovations.

#### Screening—Prioritizing Interventions and Assessing Coverage

Ideas were screened through criteria such as “Is it...desirable?” “...different?” “...feasible?” and “Will the idea move us beyond current best practice?” In design meeting 3, participants rated each intervention concept based on potential impact and degree of understanding or knowledge for implementation. Using this 2 x 2 matrix, intervention concepts could be rated high impact and high knowledge (implemented relatively easily to good effect), high impact and low knowledge (could have a positive effect but require further development), or low effect with or without high knowledge (screened out because of little or no expected impact). Intervention concepts classified as high impact but low knowledge became prototyping candidates owing to their potential for teaching us the most about particular interventions employed as part of a peer production knowledge network such as C3N.

**Objectives for design meetings.**

Design meeting 1 objectives:

1. Collaborative chronic care network (C3N) design meeting participants:
  - Meet and develop a level of comfort and familiarity with each other
  - Develop an appreciation for the broad range of expertise, experience, and approaches that each brings to the design process
  - Develop a shared understanding of the common purpose of the C3N
  - Understand the phase 0 design process
  - Develop a shared initial vision of the final C3N
  - Understand how their work fits into the C3N
2. Develop a clear articulation of the problem(s) that the human-centered design should address
3. Understand the health care ecology model
4. Give feedback to a proposed set of specific characteristics (for patients, families, clinicians who are part of networks, and researchers) that will be sampled for during the human-centered design process
5. Develop predictions of what the end users will say about their needs

Design meeting 2 objectives:

1. Introduce and reintroduce C3N participants to each other
2. Obtain input on a refined vision, purpose, values and principles, and metrics
3. Screen and elaborate a proposed initial system driver diagram
4. Screen and elaborate information, technical and experience architectures for the patient-facing portions of the design
5. Identify possible studies and prototypes

Design meeting 3 objectives:

1. Align participants around the C3N system driver diagram
2. Rate the secondary drivers as to their contribution or importance to desired system outcomes
3. Ensure that potential prototypes or work products for the next phase of the project sufficiently cover the highly rated components of the system driver diagram
4. Prioritize the prototypes or work products for the next project phase based on those that will advance the improvement and research and development efforts
5. Begin to scope the required effort, team composition, and other resources for a number of highly rated prototypes or work products

## Results

### Participants

The design process began with a relatively small team of 25 members. By actively reaching out to more participants and inviting them to “make a difference” and “be part of the solution,” we increased the number of people involved over the design phase to 150 people (9 C3N team, 28 clinicians, 9 designers, 6 informatics experts, 11 patients, 5 parents, 54 collaborators, and 27 project and research staff), exchanging over 1700 posts and messages on the private social media site. There were 8 clinicians, 3 patients, 3 parents, 2 staff, and 18 C3N team members and collaborators at design meeting 1; 6 clinicians, 3 patients, 2 parents, 6 staff, and 18 C3N team members and collaborators at design meeting 2; and 7 clinicians, 2 patients, 0 parents, 3 staff, and 12 C3N team members and collaborators at design meeting 3. All work teams that formed had representation from patient or family, clinician, and researchers stakeholder groups.

### Observation

#### Environmental Scan

Interviews with thought leaders provided the following C3N design imperatives:

- Design for all stakeholders at once—not separately for patients, clinicians, and researchers.
- Technology is only a means to an end. The focus of design must be on enabling people to gracefully achieve their goals, with appropriate technology deployed in service of those goals.
  - Notwithstanding the above, an upgradeable set of modular technologies is likely to prove to support system evolution.
- Design with acknowledgement of health care as a service that is coproduced, not a product to be delivered. This shifts the paradigm from health care as a transaction to health care as shared work [23].
- Design to enable large, diverse groups of people to identify and test many solutions to many problems. No one person and no one solution will transform care, outcomes, and cost.
- C3N leaders must empower others to achieve common aims under uncertain conditions. This entails fostering a rapid learning ethos and an embrace of failure in service of learning.

Our environmental scan uncovered 64 people, organizations, products, or services that provided inspiration for parts of the C3N. These included 5 blogs, 10 information clearinghouses, 3 design firms, 4 potential funders, 8 experts or innovators or innovations, 15 networking or community platforms, 3 stakeholder representatives, 4 information technology innovations, and 12 thought leaders. Examples include commons-based peer production models (eg, Linux, Wikipedia, TripAdvisor, Slashdot, and Science Commons); patient communities (eg, PatientsLikeMe, Crohnology, CureTogether.com, and e-patients.net); crowd-sourcing platforms such as Innocentive and Many Eyes; and QI and research collaboratives such as the Northern New England Cardiovascular Disease Study Group and the Children’s Oncology Group.

We also recognized challenges. Unlike Wikipedia or TripAdvisor, medicine has inherent power and knowledge differentials and regulatory and oversight constraints. Developing the right mix of incentives to engender collaborative behavior is challenging, as is attracting individuals to contribute and foster contributions. A free-for-all where everyone’s opinion is equal risks introducing and propagating harmful ideas and suggestions.

**Purpose, vision, values, and principles for the collaborative chronic care network (C3N).**

Creating a C3N

1. Purpose:
  - To enable patients and families, clinicians, and researchers to work together to create a *Collaborative Chronic Care Network* for inflammatory bowel disease (IBD) that transforms the outcomes and experience of illness and care, spawns innovations, and accelerates discovery and the application of new knowledge. Working with ImproveCareNow, we will design, create, and test new approaches to transforming the system of chronic illness care for IBD. By (date), the project will have produced working prototypes of components of the new system that can improve the outcomes, process, and experience of care and increase the production of innovations in care delivery and new knowledge.
2. Vision:
  - To be healthier together
  - Patients, families, clinicians, and researchers all have the same goal when it comes to chronic disease—for those affected to be healthy and live gracefully with a condition that they didn’t ask to have
  - The C3N will enable ImproveCareNow to become a collaborative innovation network—a community with shared purpose, values, tools, and technologies (both human and digital) to enable patients, families, clinicians, and researchers to share responsibility for achieving dramatically better health for children and adolescents with IBD
3. Values and principles (How we behave in the community and act toward one another):
  - Hope and compassion (to cause the enthusiasm, curiosity, and the will to solve problems)
  - Privacy (must be data literate to participate, patients own their data: have rights of possession, use, and disposal)
  - Trust (individuals demonstrate credibility, information is credible; scholarly norms for attribution; openness)
  - Shared responsibility for outcomes—we all have the responsibility to improve the health of the entire community (the entire population of patients with IBD)—you can make a difference, and you are expected to
  - Urgency and daring to create and try new ideas
  - Creativity and innovation (to generate and test new solutions)
  - Self-determination (agency)
4. Descriptions of keywords:
  - Collaborative—patients or families, clinicians, and researchers engaged as partners in a shared task.
  - Community—a distributed, voluntary organization that is interdependent, has shared responsibility, and is greater than the sum of its parts.
  - Shared purpose—improve the health of the entire IBD community
  - Common values—compassionate, safe, trust (privacy and credibility), open, self-determination, or agency
  - Flexible set of tools—human (quality improvement, leadership training, motivational interviewing, social networks, and incentives) and information (asynchronous communication, social media, network analysis, data mining, and multimedia) technologies

#### Goal-Directed Design

The goal-directed design method is presented elsewhere [16]. Personas representing all key stakeholders—patients, parents, physicians, nurses, and researchers—were created. Overall, the personas and scenarios enabled the design participants to maintain a focus on key users and how they might interact with the new system. The main contribution of this method was to keep the focus of the design on people—patients, parents, clinicians, and researchers—and on helping people meet their goals rather than to focus on the tasks required to meet these goals. For example, in one scenario, the patient persona, Bianca, is connected by her nurse, Vicki, to other patients on a virtual platform, where she overcomes her sense of isolation by sharing experiences with others similar to herself. In this case, the design was in response to Bianca’s goal to avoid isolation and remain connected to others and to Vicki’s goal of making sure patients have the support necessary to thrive. Although important tasks or features are implied in this scenario (eg, identity authentication, secure messaging, and community moderation), these were purposefully tabled to be addressed later in the design process.

### High-Level Outcome Measures

Including all relevant stakeholders as codesigners enlarged the discussion of relevant outcomes beyond traditional clinical measures. By focusing on people and their goals or needs and by insisting that the system must meet the needs of all people, we were able to arrive at a set of measures that reflected the multistakeholder perspective. The following system performance measures were proposed and approved:

- Participation, engagement, and interaction among all types of users as measured by attendance at webinars, monthly calls, and community conferences, as well as contributions of data and ideas.
- Health outcomes (eg, steroid-free remission and improved quality of life) as measured by physician global assessment.
- Reliability and effectiveness of chronic illness care (eg, more appropriate medication use and disease activity monitoring) as measured by the degree to which a bundle of clinical interventions were delivered as part of clinical care.
- Self-management, as measured by self-report of adherence.
- Production of new knowledge and discoveries as measured by research products, including grants, abstracts, presentations, and publications.

### Synthesis

#### Concept Design and Key Driver Diagram

Representatives from all stakeholder groups cowrote the purpose, vision, values, and principles for the C3N (Textbox 2) during design meeting 1, and the KDD (Figure 2) during design meeting 2. Taking a multi-stakeholder perspective forced the design team to consider the new system not as a system for doctors or a system for patients, but rather as a system for people. This, in turn, allowed ideas from outside of health care to be brought to bear in the concept development.

#### Generating Ideas and Scenarios

Design meeting 2 also resulted in ~140 potential innovation ideas. We deduplicated and combined the ideas into 33 unique potential innovations.

### Priority Setting

During design meeting 3, a total of 20 intervention concepts were rated as having high impact and high understanding or knowledge. These were interventions that ImproveCareNow was either doing currently or else were sufficiently specified so that no further design or testing was necessary. These should simply be done. There were 13 intervention concepts rated as having high potential impact and low understanding or knowledge about how to implement. These intervention concepts, listed in Textbox 3, were selected for further development and testing.

**Intervention concepts prioritized for further testing, based on high ratings on potential impact and low ratings on understanding or knowledge.**

Mentoring in inflammatory bowel disease (IBD) clinic

Leadership training

Privacy education

Facebook connector app and community building

Branding ImproveCareNow as a collaborative chronic care network (C3N)

Model care or quality improvement metric explorer

Android device—gateway to C3N

Virtual camp oasis

Self-management support curriculum

Open-source practice Wiki

Patient driven n=1 trial

Restructured IBD education day

Patient interface—virtual C3N

## Discussion

### Principal Findings

Our codesign process resulted in a community of over 100 people willing and able to self-organize to pursue a shared overall concept design for the ImproveCareNow C3N, a logic model for bringing about this system, and 13 potential innovations likely to increase awareness and agency, make it easier to collect and share information, and to enhance collaboration. Developing and testing these potential innovations to determine the degree to which they could collectively bring about the C3N were the actionable next steps for ImproveCareNow.

It is not intuitive that thousands of people could self-organize and collaborate to achieve shared aims. But we see they do across many industries. In addition to Wikipedia and other examples uncovered in our environmental scan, more recent examples such as AirBnB, Uber, Lyft, and crowd funding sites such as KickStarter and GoFundMe affect the lives of more and more people. The C3N design is a way to translate peer production to health care.

Distributed networks are especially relevant to children with chronic diseases that the NIH identifies as rare diseases [24] because no single health center has a sufficient number of patients to produce generalizable knowledge [25]. This state of affairs can result in a slow pace of knowledge acquisition and outcome improvement. Networks are also of growing importance to clinicians to support collaborative learning and application. Networks of patients and the rise of the e-patient movement (eg, Patients Like Me, Association of Cancer Online Resources, Crohnology, and Society for Participatory Medicine) have enabled patients to collect their own data for research and to support one another. But the potential of these networks to impact the overall chronic care system is limited because they operate in a siloed manner.

There is growing awareness that health care is a coproduced service—that professionals and patients create value through collaborative interactions [23]. Traditionally and appropriately, the focus has been on interactions within each clinical encounter [26]. By enlarging the focus, considering one-to-many and many-to-many interactions, and applying peer production principles, the C3N design recognizes the value of networks in health care. Fjeldstad and colleagues suggest collaborative networks share a common architecture, including actors who have the motivation and ability to self-organize; a commons where resources are created and shared; and structures, protocols, and processes that facilitate multi-actor collaboration [11]. C3N design intervention concepts can be viewed through this Actor-Oriented Architecture lens.

We conceived of the C3N as a health care system in which patients (and their families), providers (physicians and other clinicians), and researchers could collaborate, at large scale, to achieve shared aims. By codesigning with representatives of all stakeholder groups, we were able to translate this idea into a design concept, including a set of measures, a logic model, and a set of innovations that could be tested together to achieve the goal of improving care, spawning innovation, and accelerating research. The C3N model challenges the dominant chronic illness care paradigm that views patients as objects on which to intervene, structures care around episodic one-to-one patient-physician interactions, and assumes an inherent power differential based on knowledge. The C3N is designed to engage patients as coequals in care delivery, designing innovations, and research; make learning continuous; and level the knowledge gradient.

### Challenges Encountered

We encountered several challenges during this design phase. Because a C3N had never been created before, we did not know what the end product ought to be, and this was frustrating to some stakeholders who wanted to know what the answer was. Over the course of the design process, most stakeholders came to realize that there was no predesigned product and that the point of the codesign process was to come up with this answer. Another challenge was managing expectations of how transformative the changes would be. Some stakeholders were nervous that the design would be too much of a change, whereas others feared the opposite. We regularly introduced the topic of change and attempted to calibrate expectations, in part, by reiterating that the codesign process itself would ensure that the final product was acceptable to the community. A third challenge was the need to translate across stakeholders so that a common perspective and even a common language emerged. Words like *community* and *social network* are used in common parlance but have specific scientific meanings that may be different from their connotations.

### Limitations

Because a C3N had never been made before, there was no way to know in advance what steps to follow to bring it about. We developed, rather, a collaborative team of more than 100 diverse stakeholders aligned around a common goal and with a common plan for testing our way into this new system. This team was able to identify user needs and generate a sufficient set of novel ideas that could be potentially transformative for ImproveCareNow [21].

The clinicians and patients with whom we worked are likely to be systematically unrepresentative of the population, having relatively high levels of skills, insights, or resources. This set of conditions risks creation of a design that would work only for these users. Our rationale for this strategy is based on von Hippel’s theory of lead-users [27] that posits that in the case of a new product or product category, most users will not have the real-world experience necessary to contribute to its development. Lead users are those whose current strong needs will become general in the near future. They often attempt to fill their needs by creating novel solutions. Accordingly, we identified and worked with lead users in the codesign process. In addition, we guarded against a narrow design through the use of personas and scenarios developed through goal-directed design, which offers design targets more representative of potential users with fewer advantages.

The generalizability of the C3N design is unknown. Although this design was built for IBD, the noncategorical approach to chronic illness care [28] and the chronic care model [26] both suggest that there are common problems faced by people and common processes necessary for good clinical care across conditions. This would argue for generalizability. However, not all chronic disease is like IBD: when patients are in remission, patients with IBD feel well and may forget about the disease. Other illnesses such as diabetes or cystic fibrosis require relentless attention to care. We intend to test the C3N design in other conditions.

Finally, the absence of formal feedback from our codesign participants limits our ability to understand how acceptable different users found the process.

### Conclusions

Our current health care system cannot achieve the results we need. Incrementally improving the current system is not enough, but designing a new system is a daunting task. Our experience suggests that codesigning with representatives from all relevant stakeholders, using the idealized design process, can result in a potentially transformative design for the chronic care delivery system.

## Abbreviations

C3N: collaborative chronic care network
IBD: inflammatory bowel disease
KDD: key driver diagram
LHS: learning health system
NIH: National Institutes of Health
QI: quality improvement
